# The Effect of Credibility-Related Design Cues on Responses to a Web-Based Message About the Breast Cancer Risks From Alcohol: Randomized Controlled Trial

**DOI:** 10.2196/jmir.1097

**Published:** 2009-08-25

**Authors:** Peter R Harris, Elizabeth Sillence, Pamela Briggs

**Affiliations:** ^2^School of Psychology and Sports SciencesNorthumbria UniversityNewcastle upon TyneUK; ^1^Psychology DepartmentUniversity of SheffieldWestern BankSheffieldUK

**Keywords:** Alcohol, breast cancer risk, health risk information, Internet, Web design

## Abstract

**Background:**

Internet sites typically contain visual design elements that are unrelated to the quality of the health information presented but that could influence credibility judgments and responses to health advice. To assess the effects of such design elements, or credibility cues, experimentally, we exposed women with different levels of weekly alcohol consumption to a website containing high quality but unpalatable information about a related health risk (breast cancer). The information was presented alongside either positive or negative credibility cues unrelated to information content.

**Objectives:**

We explored four research questions: (1) Did the cues influence how the women engaged with the site? (2) Did they influence how the women responded cognitively and emotionally? (3) Did they influence whether the women subsequently acted on the advice? (4) Did the impact of the cues vary with how much alcohol the women reported drinking?

**Method:**

A total of 85 women were randomly assigned to view one of two versions of a website containing the same high-quality content but different cues. One version had positive credibility cues (trustmarks), the other had negative ones (adverts, pharmaceutical sponsorship, and a donation button). Objective measures included visual attention (using eye-tracking equipment), time studying the material, and recall. Subjective measures included cognitive and affective responses and intention to change. Measures of subsequent behavior were taken 1 week later.

**Results:**

First, the cues did not affect how long the women spent on the site or how long they spent reading the text. However, women in the negative cues condition spent more time looking at a donation button than those in the positive cues condition spent looking at a TRUSTe seal (β = −.43, *P* < .001) but less time looking at a logo (β = .43, *P* < .001) or at certain other features of the site. Those in the negative cues condition also recalled more site content (β = −.22, *P* = .048). Second, there were no effects of the cues on any of the measures of cognition, affect, vulnerability, or intentions. However, third, at follow-up, the positive cues had promoted greater alcohol reduction than the negative cues among those women who had previously reported drinking more heavily (β =  −.22, *P* = .02). So, fourth, the responses to the cues did vary with how much alcohol the women typically drank.

**Conclusions:**

Content-irrelevant images and logos can influence the behavioral response to quality health-risk information. These effects may be subtle, changing with time.

## Introduction

The Internet has become an important source of health information and advice, with between 40% and 80% of those with Internet access in the United States and Europe using it for health care purposes [1,2]. However, the quality of the available information is highly variable. For example, in a 2002 meta-analysis, Eysenbach and colleagues noted that 70% of website reviews expressed concern about the quality of the health-related information provided on the Internet [3]. In the face of such variable quality, how do health consumers decide whether or not to trust the information and advice they find online?

Briggs and colleagues addressed this issue in a series of studies of trust in eHealth that led to the development of a staged model of trust and a set of guidelines [4-6]. They found that users rapidly rejected health sites on the basis of superficial cues capable of influencing consumer trust. These included advertising, complex layout, inconsistent design, or the presence or absence of reputable brand markers.

Sillence et al [5,6] noted that a great deal of high-quality health information was lost to the consumer through this process and that drug company sites in particular—often highly rated by health experts in terms of health content [7]—were frequently rejected because of the presence of commercial cues.

Health consumers, then, do not always choose the best-quality health sites or follow the best advice. Indeed, they can show a marked reluctance to trust advice they perceive to be inconsistent with their important prior beliefs, even displaying a “defensive” response to such information [8-11] that can take the form of close critical scrutiny and subsequent rejection of good-quality information [12,13]. As a result, those most at risk can be the most averse to change and may be the most difficult to persuade. Clearly, given this propensity to respond defensively, it is counter-productive to present consumers with cues that might inadvertently trigger a negative response to good health advice.

This is particularly problematic in the eHealth context, where consumers can often find conflicting advice and are able to navigate to websites that tell them what they would prefer to hear. In one study, for example, a population struggling to modify their drinking behavior sought pro-drinking rather than anti-drinking material when left free to choose via the Internet [14]. In our own study, we are interested in the fact that Internet material often contains visual design elements, or cues, that are unrelated to the quality of the health information presented but that could be used to influence credibility judgments about the site and that may also subsequently influence acceptance and adherence to health advice. What happens to an individual who spends time reading important high-quality health advice on a website that coincidentally displays negative credibility cues (such as a drug company site containing high-quality health information alongside advertising)? Can these content-irrelevant cues affect acceptance or rejection of important health advice?

We present an experimental study in which women with different levels of weekly alcohol consumption were exposed to high-quality, uncongenial, Internet-based information concerning a related health risk (breast cancer). This unpalatable information was presented alongside positive or negative trust cues unrelated to the information content. We explored four research questions: First, do the cues influence direct engagement with the material on the site? We examined how long the women spent on the site, their pattern of eye movements when examining the site’s pages, and their ability to recall site content. Second, do the cues influence how they respond cognitively and affectively to the site? We examined their subsequent message acceptance, emotional and risk perceptions, and their intentions to adopt the recommended behavior. Third, do the cues influence the extent to which they subsequently act on the advice given? We examined reports of behavior at follow-up 1 week after exposure to the site. Finally, does the impact of the cues vary with how much alcohol the women typically drink? We tested whether baseline alcohol consumption moderated the effects of condition on any of the above outcome measures. That is, we tested whether any cue effects were most pronounced among those who drank most (i.e., those who might display the biggest defense motivation) [10,15].

To test these research questions we developed a two-page website containing information about the link between alcohol consumption and the risk of breast cancer. All material was based on a recent definitive study showing such a link [16]. At the time of testing this was (and in the UK largely remains) a relatively unknown association, so for most women the information on the site was novel. The text contained strong arguments that were not easily denigrated and was identical in both content and layout in both experimental conditions. Participants—women aged 18 to 46 who varied in their level of alcohol consumption—were randomly exposed to this information on a website containing either positive credibility cues (i.e., cues that had been positively associated with credibility in previous research [6]) or negative credibility cues (also based on previous findings [17]). The cues were selected to be unrelated to the content of the site. In summary, the aim of this study was to assess whether design-based credibility cues promote acceptance or rejection of important health advice.

## Methods

### Participants

The sample consisted of 85 women, all regular Internet users and mainly students of psychology (mean age = 22.9 years, SD = 6.5 years). Of these, over a quarter (n = 22) reported drinking more than the UK Government’s recommended limit of 14 units of alcohol per week. (In the UK, a unit of alcohol is 8 g, which is approximately half a pint of beer, a standard measure of spirits, or a glass of wine.) All participants were paid £5.

### Design

The study had a between-participants experimental design, with one independent variable: participants were randomly assigned to the positive (n = 42) or negative (n = 43) cues conditions. The experiment had a prospective component, with a follow-up after 1 week.

### Materials and Measures

#### Pre-Manipulation Measures

Along with baseline alcohol consumption, we also measured age, sex, Internet use, attitudes toward alcohol [15], dispositional optimism [18], self-esteem [19], and breast cancer risk [20,21] (Table 1). Baseline alcohol was measured using the procedures employed by Harris and Napper [15], adapted from Dawson and Room [22]. Participants were asked how much alcohol they had consumed in the last 7 days, how much they consumed in a typical week, and how much they had consumed in the last 24 hours. Responses were given in terms of pints of beer/lager/cider, shots, glasses of wine, and bottles (e.g., of beer), with illustrative examples of all types, and later translated into units of alcohol by the experimenter. Del Boca and Noll [23] have shown that self-reported alcohol consumption is at least as accurate as biomarker data for measuring drinking patterns in adult general populations. Reports of alcohol consumption in the past week and in a typical week were strongly correlated (Pearson *r* = .70, *P* < .001) and were combined into a mean score for analyses. Alcohol consumption varied from 0 to 42.5 units (mean = 10.48, SD = 9.41).

#### Health Message

The health message was on two Web pages and contained information about the link between alcohol consumption and the risk of breast cancer. The text of the Web pages was closely based on that used by Harris and Napper [15]. The information was taken from a press release from Cancer Research UK [24] and a newspaper article [25]. All statements it contained were true.

#### Cues

Two different designs of the Web pages were created (see Multimedia Appendix 1 and 2). Both designs contained exactly the same health message but varied in terms of the presence of adverts, sponsors, and donation requests. The negative condition contained adverts, pharmaceutical sponsorship, and a donation button, whereas the positive condition contained a TRUSTe seal and a Health On the Net foundation (HON code) certificate. Websites can apply to be accredited by these two organizations if their site meets a number of trust indicators, including privacy, transparency, author qualifications, attribution, and justifiability. These cues were chosen from the list of credible and non-credible cues elicited in earlier phases of the research program [5,6] and were selected for their lack of direct relation to content. The size and location of these different design features were constant across both conditions.

#### Eye-Tracking Measures

The SensoMotoric Instruments iViewX Eye Tracking System (SensoMotoric Instruments GmbH (SMI), Teltow/Berlin, Germany) was used to record the eye movements of participants as they were viewing the Web pages on a desktop PC (1.2 GHz Pentium 4 with 256 Mb memory and a 17 inch LCD monitor). In accordance with the eye-tracking analysis software, the different design features of interest on each Web page were defined as object areas; 15 such objects were identified (Table 2). For example, object 1 was defined as the donation button in the negative condition and the TRUSTe seal in the positive condition. Some of these objects (e.g., page last updated) were common to both conditions. For each condition, the percentage of time spent looking at each object area was calculated (Table 2).

#### Post-Manipulation Questionnaire

After viewing the message, participants completed the post-manipulation questionnaire. This opened with the manipulation check items, comprising a measure of mood [15] followed by the trust scale developed in an earlier phase of the research program [17]. The trust scale has four factors:

1. information access: eight items (e.g., “The site told me most of what I needed to know”)
2. personalization: eight items (e.g., “It felt like the advice was tailored to me personally”)
3. credibility through impartiality: four items (e.g., “The advice appeared to be impartial and independent”)
4. credibility through design: four items (e.g., “The site had a professional design”)

Responses were given on a 5-point scale from “strongly disagree” to “strongly agree.”

The subjective measures comprised the following:

1. cognitive responses indicating acceptance of the message: three items (e.g., “I believe that drinking alcohol increases the chances of women developing breast cancer) rated on a 7-point scale from “strongly disagree” to “strongly agree”
2. negative affective responses to the message: six items (e.g., “The material on the website made me feel…”) rated on a 7-point scale from “not at all anxious” to “extremely anxious”
3. perceived vulnerability: three items (e.g., “How likely do you think you will be to get breast cancer as a result of your current level of alcohol consumption) rated on a 10-point scale from “impossible” to “extremely likely”
4. intentions to cut down on alcohol: two items (e.g., “I intend to reduce my alcohol consumption in the next 7 days by at least 2 units”) rated on a 7-point scale from “definitely do not intend to” to “definitely intend to”

Items were taken from previous studies (e.g., [15,26]). All had satisfactory reliabilities (Cronbach alpha from .72 to .98).

The questionnaire closed with a test of recall of message content (three items; e.g., “According to the report how many deaths would be avoided annually in Britain if women stopped drinking?”). Each correct answer scored 1.

#### Follow-Up

After 1 week, participants received a brief follow-up questionnaire by email, measuring reported alcohol consumption over the previous 7 days and containing the measures of intentions, vulnerability, cognitive response (one item), and affective response (three items) from the post-manipulation questionnaire (Cronbach alpha from .70 to .97). The questionnaire was designed to be brief in order to maximize response rate.

### Procedure

Participants were tested individually. Upon arriving at the laboratory, they were told that the study involved an evaluation of health information on the Internet. After completing the pre-measures, they were asked to sit comfortably in front of the eye-tracking monitor while the researcher calibrated the system. Each participant was told that she was about to see a website consisting of two pages. They were instructed to imagine that they had found the website by following a link from a search engine and to look at and visually examine the website as they would any site they had found in this way. They were told that they could spend as little or as much time as they wanted on the website. This phase of the study ended when the participant clicked on a link at the bottom of the second Web page. They then completed the post-manipulation measures. Participants were sent the follow-up measures by email approximately 7 days later.

### Statistical Analysis

One-way analysis of variance (ANOVA) with randomized condition as the between-participants independent variable or two-step hierarchical regression analyses were used to test for differences in pre-manipulation measures or other variables between the groups. An alpha level of *P* = .05 was set for all analyses.

## Results

### Randomization and Manipulation Checks

One-way analysis of variance (ANOVA) with randomized condition as the between-participants independent variable revealed no significant differences between the groups (Table 1; maximum F_1,56_ = 1.52 [Halls breast cancer risk index], *P* = .22, eta^2^ = .03). Thus randomization appears to have been successful.

Consistent with the intended manipulation, participants in the positive cues condition trusted the site more on the two credibility trust factors, seeing the site as being higher in credibility through impartiality (F_1,82_ = 4.74, *P* = .03, eta^2^ = .06) and design (F_1,82_ = 4.92, *P* = .03, eta^2^ = .06; see Table 3). Thus, the manipulation appears to have been successful. Participants did not differ significantly on the access or personalization trust factors or on mood (maximum F_1,70_ = 3.31 [positive mood], *P* = .08, eta^2^ = .04).

**Table 1:** Mean pre-manipulation measures by condition group^a^

	Negative Cues (n= 43) Mean score (SD)	Positive Cues (n= 42) Mean score (SD)
Baseline alcohol (units)^b^	10.03 (9.10)	10.95 (9.80)
Attitudes toward alcohol^c^	4.23 (0.95)	4.25 (0.92)
Self-esteem	19.33 (3.73)	20.05 (3.87)
Dispositional optimism	14.21 (4.60)	14.56 (4.74)
Breast cancer risk (Harvard risk calculator)	2.56 (0.85)	2.63 (0.80)
Breast cancer risk (Halls risk calculator)	16.72 (3.36)	18.02 (4.72)

^a^Higher scores indicate more alcohol consumption, favorable attitudes, and greater self-esteem, optimism, and risk.

^b^Alcohol measured in UK units (one unit is 8 g).

^c^Attitudes were measured on a 7-point bipolar scale.

### Objective Measures

Unless stated otherwise, the remaining data were analyzed using two-step hierarchical regression analyses. This allowed us to analyze the effects of baseline alcohol consumption as a continuous variable and to assess the effects of the predictors in combination as well as individually. Where an individual predictor was significant, we report it (β) below; otherwise, in the interests of space, we report only the statistics for the predictors in combination, both in terms of significance and effect size (*R* ^2^). At step one, we assessed the main effects of condition (negative cues = 0; positive cues = 1) and baseline alcohol consumption; at step 2, we added the condition × consumption interaction. In accordance with the recommendations of Aiken and West [27], the independent variables were mean centered.

There were no significant overall effects of the predictors on the amount of time participants spent on the site (F_3,84_   < 1, *R* ^2^ = .02) or on how long they looked at the text (F_3,73_ = 1.08, *R* ^2^ = .04). None of the individual predictors (condition, alcohol consumption, or the interaction) approached significance for these dependent variables. However, there were significant main effects of condition on patterns of eye movement to certain features of the site (see Table 2). Women in the negative cues condition spent more time looking at the donation button than those in the positive cues condition spent looking at the TRUSTe seal (β = −.43, *P* < .001). In contrast, they spent less time looking at the logo (β = .43, *P* < .001), menu 1 (β = .38, *P* < .001), and when the site was last updated (β = .27, *P* = .02). There was a significant main effect of condition on recall (see Table 3), with those in the negative cues condition having significantly better total recall (β = −.22, *P* = .048). Baseline alcohol consumption did not affect eye movement or recall. No interaction was significant.

**Table 2:** Percentage of time participants spent visually examining objects on the website, by condition^a^

Object^b^	Negative Cues (n= 43) % of time (SD)	Positive Cues (n= 42) % of time (SD)
1. Donation/TRUSTe seal	5.03 (4.33)	1.80 (2.29)
2. Logo	5.92 (4.07)	12.82 (9.39)
3. Menu 1 (“You are here”)	5.00 (4.13)	9.80 (7.14)
4. Menu 2 (“Health issues”)	2.75 (2.86)	3.42 (3.31)
5. Advert/HON code	1.44 (1.80)	0.95 (1.68)
6. Page last updated	0.00 (0.00)	0.18 (0.50)
7. Sponsor – Pharmaceutical/NHS	0.25 (0.91)	0.34 (1.34)
8. Photo	1.50 (1.75)	1.39 (1.62)
9. Alcohol and breast cancer subtitle	1.50 (1.82)	1.45 (1.55)
10. Next page link	0.08 (0.28)	0.39 (1.67)
11. Text paragraph 1	6.31 (4.44)	6.32 (4.80)
12. Text paragraph 2	10.47 (7.13)	7.47 (7.05)
13. Text paragraph 3	3.40 (3.83)	2.82 (4.14)
14. Application window^c^	57.25 (28.27)	62.03 (26.00)
15. Entire screen^d^	60.58 (28.15)	65.16 (25.80)

^a^Standard deviations are given in parentheses.

^b^ Objects with different cues (i.e., Advert/HON code and Pharmaceutical/NHS sponsor) are negative cue/positive cue.

^c^ Application window refers to Web browser.

^d^ Entire screen refers to everything visible on the monitor (i.e., application, borders, and task bar).

### Subjective Measures

Condition did not affect any of the measures. No interaction was significant. Level of baseline alcohol consumption did not affect cognitive responses to the material, but higher levels of alcohol were associated with more negative affect (β = .48, *P* < .001), higher perceived vulnerability (β = .31, *P* = .004), and stronger intentions to reduce alcohol (β = .37, *P* = .001).

**Table 3:** Other dependent variables, by condition^a^

	Negative Cues (n= 43) Mean score (SD)	Positive Cues (n= 42) Mean score (SD)	Total (n= 85)
**Manipulation checkitems**			
Trustfactor 1 (access)	30.05 (4.60)	31.00 (3.56)	30.51 (4.13)
Trustfactor 2 (personalization)	16.63 (3.70)	16.88 (2.90)	16.75 (3.31)
Trustfactor 3 (impartiality)	12.33 (2.10)	13.51 (2.86)	12.90 (2.55)
Trustfactor 4 (design)	11.56 (2.36)	12.93 (3.24)	12.23 (2.89)
Positive mood	2.90 (1.03)	2.47 (1.05)	2.71 (1.05)
Negative mood	1.00 (1.41)	1.09 (1.53)	1.04 (1.46)
**Outcome measures**			
Recall^b^	1.07 (0.93)	0.66 (0.85)	0.87 (0.91)
Intentions to cut down alcohol	2.60 (1.64)	2.88 (1.81)	2.79 (1.73)
Cognitive responses	4.29 (1.12)	3.90 (1.12)	4.09 (1.14)
Negative affective response	3.54 (1.34)	3.58 (1.66)	3.56 (1.50)
Perceived vulnerability	2.31 (1.09)	2.44 (1.16)	2.38 (1.12)
Baseline alcohol consumption^c^	10.03 (9.10)	10.95 (9.80)	10.48 (9.41)
**Follow-up**	(n= 36)	(n = 39)	(n = 75)
Alcohol consumption^c^	10.63 (9.48)	9.68 (9.36)	10.13 (9.37)
Belief in link	4.53 (1.60)	4.64 (1.31)	4.59 (1.44)
Intentions to cut down alcohol	2.60 (1.64)	2.98 (1.81)	2.79 (1.73)
Negative affective responses	3.39 (1.90)	3.72 (1.96)	3.56 (1.93)
Perceived vulnerability	2.18 (0.87)	2.34 (1.15)	2.26 (1.02)

^a^Higher scores indicate more trust, more positive/negative mood, better recall, stronger intentions, more acceptance of the message, more negative affect, higher perceived vulnerability, and more alcohol consumption.

^b^Maximum possible recall is 3.

^c^Alcohol measured in UK units (one unit is 8 g).

### Follow-Up

**Figure 1:**
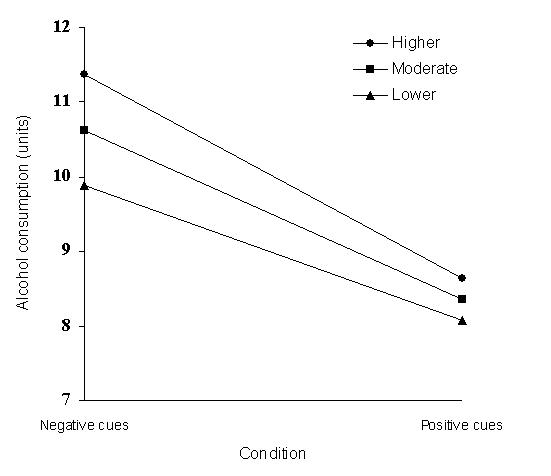
Simple slopes for the interaction between condition and baseline alcohol consumption on alcohol consumption at 1 week follow-up; simple slopes have been calculated at mean (moderate consumption), +1 standard deviation (higher consumption), and −1 standard deviation (lower consumption) levels of baseline consumption

A total of 75/85 (88%) participants responded to the follow-up questionnaire. These women did not differ significantly from nonresponders on any of the randomization or manipulation check measures taken at post-manipulation (maximum F_1,82_ = 1.1 [trust factor 3], *P* = .30, eta^2^ = .013). There was no significant association between condition and responding to the follow-up (χ^2^_1,85_ = 1.71, *P* = .33). There was a significant main effect of baseline alcohol consumption (β = .72, *P* < .001) but not of condition (β = −.12, *P* = .17) on reported alcohol consumption over the previous 7 days. More importantly, however, there was a significant interaction between condition and baseline consumption on reported alcohol consumption (β = −.22, *P* = .02), indicating that how much the women reported drinking at the time they were exposed to the website determined the extent to which they responded to the cues by subsequently reducing their drinking. Inspection of the simple slopes [27] indicated that at higher and moderate levels of baseline alcohol consumption, the positive cues led to greater reductions than did the negative cues (Figure 1). Overall, those in the positive cues condition reported an average decrease of 1.3 units, whereas those in the negative cues condition reported an average increase of 0.6 units.

As for the post-manipulation, higher levels of baseline alcohol in the follow-up were associated with more negative affect (β = .52, *P* < .001), greater perceived vulnerability (β = .39, *P* = .001), and stronger intentions to cut down on alcohol (β = .26, *P* = .04). Moreover, those who drank more at baseline now perceived the evidence linking alcohol and breast cancer to be significantly weaker (β = −.24, *P* = .02), thus perhaps showing the first signs of a defensive reappraisal of the message. Indeed, condition interacted with baseline alcohol consumption on this measure of belief in the evidence (β = .38, *P* = .001), with the effect being more pronounced in the positive than in the negative cues condition.

## Discussion

Did the cues influence engagement with the material on the site? We examined how long the women spent on the site, their pattern of eye movements when examining it, and their recall of site content and found that, whereas the cues did not affect the overall time the women spent on the site or how long they spent reading the text, they did influence where the women directed their gaze and how much they subsequently recalled. Participants paid more attention to certain negative cues, such as the donation button, than to positive cues in the equivalent location, and those in the negative cues condition were subsequently more accurate in their recall of information, suggesting that they paid closer attention to the site content.

Did the cues influence how the participants responded cognitively and affectively? No. We found no effects of the cues on any of the explicit ratings of cognition, affect, vulnerability, or intentions—at least not initially (see below). However, those who drank more reported more negative affect and vulnerability and expressed stronger intentions to cut down on their drinking, effects that persisted at follow-up. This suggests that the material had been persuasive, even among the heavier drinkers. Interestingly, these effects occurred even though drinking level had not influenced how the women examined the site visually or their recall of its contents.

Did the cues influence the extent to which the women subsequently acted on the advice given? Yes, the important influence of credibility cues was seen most clearly at follow-up, where the positive cues promoted greater alcohol reduction than did the negative cues among those who had previously reported heavier drinking. Thus, the answer to our final question— Does the impact of the cues vary with how much alcohol the women typically drank?—is also yes. Indeed, the cues now interacted with baseline drinking level to change the women’s belief in the evidence: specifically, women who had reported drinking the most expressed less belief in the evidence at follow-up, especially the women in the positive cues condition.

Consequently, the results of this study indicate that seemingly superficial design elements of a website can influence responses to health-risk information. As predicted, cues known to be positively or negatively associated with credibility affected engagement with the site and influenced subsequent health behavior and cognition.

We interpret these data as being consistent with findings in the broader literature on defensive responding to threatening information [8-11]. In essence, we expected the presence of positive (but non-content-related) credibility cues to bolster the message but negative cues to undermine it. We were unsure how immediate or delayed such effects might be, and it is clear that, in the short term, message content predominated. However, with time, the negative cues exerted detrimental effects by reducing the extent to which the heavier-drinking women acted on the advice. Moreover, 1 week later, the heavier drinkers also reported perceiving the evidence linking breast cancer and alcohol to be weaker, thus showing the first signs of message rejection on any of our explicit measures. Perhaps this is because, after a week of reduced consumption, they appreciated more fully the difficulties involved in trying to reduce alcohol consumption. If so, we may have captured them in the early stages of defensively re-evaluating the original website material. This might be the focus for further investigation. Indeed, at this stage there was even the suggestion that the protective effects of the positive cues may have been wearing off, as belief in the link was lower among heavier drinkers in the positive cues condition.

The findings thus present an intriguing picture of a group of participants developing their response to a website containing credible, but unwelcome, health-related information. The eye-tracking data also contribute to our understanding of how people allocate their attention to features of websites. For instance, those in the positive cues condition spent relatively little time looking at the HON code or the TRUSTe seal relative to other elements of the site, such as the logo (see Table 2). Indeed, the presence of such codes and awards has been shown elsewhere to have little effect on the credibility or retention of health information on a Web page [28], and Eysenbach and Köhler [29] noted that consumers failed to click on the HON logo when it was present on websites, despite having suggested that some form of controlling authority or an endorsement by a third party would be a helpful quality marker. There have been claims that consumer expectations of such health seals are often inconsistent with how they respond to them in practice [30]. Certainly, here participants were more inclined either to look at negative cues more than their positive equivalents or to spend relatively little time looking directly at either.

### Limitations

We have shown here (to our knowledge for the first time) that seemingly superficial credibility cues embedded in health information and advice can continue to affect responding over time. However, we acknowledge that our study has several limitations. We recruited a mainly student sample to participate in a somewhat artificial environment, under experimental conditions. We cannot rule out the possibility that some participants may have felt themselves “too young” to be at risk of breast cancer or felt obliged to accept the health message contained in the website. We tried to minimize this by explicitly instructing participants to take their time and to explore the site visually as if they had discovered it themselves through a search engine. We also ensured that the health message was aimed at young women in particular. We recognize, however, that the experimental conditions also meant that participants were forced to look at the website and therefore could not choose to “select out” sites as perhaps they would in more natural settings.

### Conclusions

Despite these limitations, these findings are potentially very significant since at-risk groups are typically the hardest to persuade [12,13]. Web-based intervention programs can also reach large numbers of heavier drinkers who might otherwise not seek help [31,32]. Our results suggest that there may be a health benefit in combining uncongenial information with positive credibility cues (although the benefits of this over the longer term remain to be researched). The implications in terms of the design are not limited to online presentation of material. Poor design features also occur in non-Internet material, for example, in patient leaflets [33], and negative design cues such as logos, sponsorship, and advertising are commonly found in these materials.

More studies of this kind are needed to explore both the immediate and longer term effects of design cues on health cognition and behavior. The message in this study was strong and persuasive and not easily rejected. It may be that the effects of negative design cues such as advertising are more immediate in evaluations of health risk messages that are weaker. Similarly, future research needs to explore what happens when sites containing credible design cues present incorrect information but noncredible sites present correct information. Longer-term follow-ups are also required, along with studies focusing on other health risk messages and populations.

Written information, often in the form of patient leaflets, has usually been seen as an adjunct to verbal messages provided by the medical profession [34] that may enhance and encourage behavior change [35]. Efforts are being made within the UK health service to evaluate traditional methods of conveying information to patients and to develop and assess new approaches [36]. The impact of design cues on subsequent behavior has implications for those involved in producing useful and effective patient information.

## Multimedia Appendix 1

Screenshot of the positive cues website

## Multimedia Appendix 2

Screenshot of the negative cues website
