# Supplementary material for: The Effect of Credibility-Related Design Cues on Responses to a Web-Based Message About the Breast Cancer Risks From Alcohol: Randomized Controlled Trial
Source: J Med Internet Res. 2009 Aug 25;11(3):e37. doi: 10.2196/jmir.1097 (PMC2762857; doi:10.2196/jmir.1097)
Supplement: Supplementary file 2 [file jmir_v11i3e37_app2.pdf]

Menopause, perimenopause, postmenopause and womens health at ProjectAWARE - Microsoft Internet Explorer provided by BT Webtop

C:\temp\liz\new\Alcnegcues1\Alcnegcues1.htm

File Edit View Favorites Tools Help

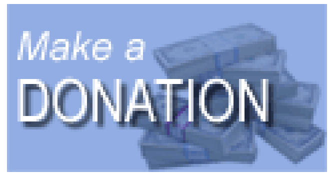

We need your support

Alcohol AWARE

You are here: Home> Alcohol issues and concerns> Breast cancer

Page Last updated January 2006

HEALTH ISSUES

- Alcohol
- Issues and concerns
- Breast cancer

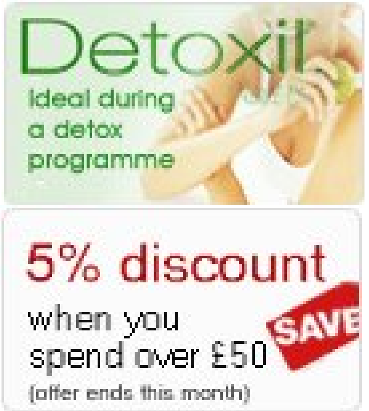

Alcohol and Breast cancer

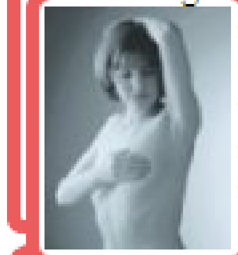

Drinking a single alcoholic drink a day increases a woman's chance of developing breast cancer by around 6%, according to a [illegible]ajor global study of woman's drinking behaviour. The new research, from Cancer Research UK and published in [illegible]he British Journal of Cancer, estimates that if women in Britain stopped drinking, 2,000 deaths from breast cancer [illegible]ould be avoided annually. Professor Valerie Beral of Cancer Research UK's cancer epidemiology unit said, "This [illegible]esearch tells us that there is a definite link between alcohol and breast cancer and the evidence suggests that the [illegible]ore a woman drinks, the greater her risk."

Past research has been inconclusive about the role of alcohol in the development of breast cancer. The sheer size of the new study, including data from around 150,000 women around the globe, allows the researchers to make the most accurate estimates ever of the risks associated with drinking. Sir Richard Doll, a co-author of the study, said: "There has been a great deal of research on whether alcohol contributes to breast cancer but until now results have been confused. For the first time we have undertaken a study large enough and detailed enough to look at the effects of alcohol reliably. When we did this we found that drinking increases the risk of breast cancer."

While women who drink regularly are at a higher risk of a number of diseases, including cancers of the throat and liver, they are at a lower risk of heart disease and stroke than are non-drinkers. Dr Gillian Reeves, who also co-authored the report, says: "The balance between the harmful effects of alcohol on breast cancer and its beneficial effects on heart disease depend on a woman's age". It is not until after the age of 65 or so that the benefits of moderate drinking become apparent and before then the risk of breast cancer is far higher than that of heart disease.

Next page>>>>>>>>

Visit Our Sponsors

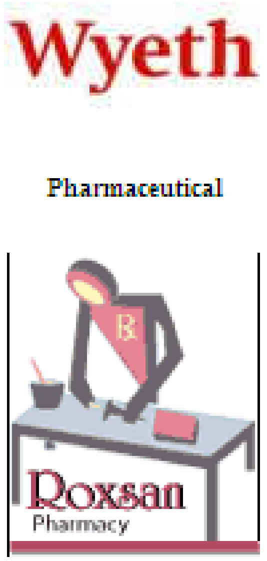

Done My Computer 100%
